# Using the Modified Apical Access Technique to Treat Peri-Implant Mucosa Defects: Description of the Technique and Three-Dimensional Quantitative Measurement of Buccal Augmented Tissue

**DOI:** 10.3390/dj12070194

**Published:** 2024-06-24

**Authors:** Norberto Quispe-López, Tiago Marques, Yasmina Guadilla, Javier Flores-Fraile, Pablo Garrido-Martínez, Javier Montero

**Affiliations:** 1 Department of Surgery, Faculty of Medicine, Dental Clinic, University of Salamanca, Campus Miguel de Unamuno, 37007 Salamanca, Spain; yguadilla@usal.es (Y.G.); j.flores@usal.es (J.F.-F.); javimont@usal.es (J.M.); 2 Faculty of Dental Medicine, Universidade Católica Portuguesa, 3504-505 Viseu, Portugal; tmmarques@ucp.pt; 3 Centre for Interdisciplinary Research in Health (CIIS), Universidade Católica Portuguesa, 3504-505 Viseu, Portugal; 4 Department of Prosthesis, Faculty of Health Sciences, Alfonso X El Sabio University, 28691 Madrid, Spain; pablogarrido86@hotmail.com

**Keywords:** dental implants, de-epithelialized free gingival graft, apical approach, soft tissue management, three-dimensional analysis, STL file

## Abstract

The importance of augmenting the peri-implant soft- and hard-tissue architecture is now widely accepted. However, while most contemporary research supports this premise, clinicians are encountering peri-implant soft tissue defects with increasing frequency, which they are therefore required to reconstruct. These complications can result from the difficulty of establishing an appropriate diagnosis and treatment plan or from suboptimal clinical situations (implant malposition, insufficient vestibular alveolar bone thickness or inadequate mucosal thickness). In this context, it is the peri-implant soft-tissue phenotype that most influences esthetic and health-related results in the short and long term. This article describes two clinical cases in which a modification of the apical access technique is presented that may be useful in clinical scenarios requiring large gains in mucosal thickness. Use of the modified bilaminar apical access with de-epithelialized free gingival graft technique showed promising results, with a significant increase in mucosal thickness and satisfactory outcomes in esthetics and peri-implant health.

## 1. Introduction

Hard and soft tissue maintenance and/or augmentation are two of the most important factors influencing the long-term survival of dental implants [1,2,3]. Today’s clinicians and researchers view the peri-implant mucosa and bone as a closely connected system of great clinical relevance [4].

Achieving functional and esthetic results with long-term maintenance is possible, provided that a meticulous diagnostic process is followed and a personalized treatment plan devised [5,6]. However, inadequate treatments leading to complications with peri-implant soft and/or hard tissue remain a problem [7]. These complications are most often caused by implant malposition (excessively buccal) and the restoration design [8].

Reconstructing the architecture of the peri-implant alveolar crest through guided bone regeneration or block grafting is a well-integrated part of the daily practice of clinicians and researchers [9,10]. In contrast, peri-implant soft-tissue management is an area in need of development, as we do not always augment the peri-implant soft-tissue phenotype, and this omission can result in short- and long-term complications [7]. To resolve these peri-implant soft tissue complications, surgical techniques have been recommended that reconstruct or augment the keratinized mucosa through the use of autologous or xenogeneic soft-tissue grafts [2,6,11].

Current evidence suggests that the peri-implant phenotype is determined by the keratinized mucosa width (KMW), mucosal thickness (MT), supracrestal tissue height (STH) and an osseous component, defined by the peri-implant bone thickness (PBT) [4,12]. If the peri-implant phenotype measures less than 2 mm, the implants may cause more discomfort during toothbrushing and there may be a greater accumulation of biofilm, resulting in an increased prevalence of mucositis and peri-implantitis [12].

While the concepts are well defined, precisely monitoring the dimensional changes that occur after placing a connective tissue graft (CTG) remains a difficult task. For many years, studies on this topic recorded changes in the periodontal/peri-implant phenotype using linear clinical measurements [13]. For example, the most common methods of assessing gingival thickness included transgingival probing [14], ultrasound [15] and radiography. However, in recent years, a significant number of publications using digital measurement methods have appeared [16,17,18]. The use of analysis software enables measurements to be taken in a repeatable manner and volumetric changes to be measured quantitatively over different periods of time, independently of the observer [19,20].

Therefore, the purpose of the present study was to present and provide a detailed description of two clinical cases with a modified version of the “apical approach” technique. The objective of this modified technique is to achieve greater gains in mucosal thickness by improving the vascularization of the peri-implant recipient bed. Additionally, a three-dimensional quantitative measurement of the buccal augmented tissue after the surgery was performed by means of intraoral scanning.

## 2. Materials and Methods

### 2.1. Case 1 

#### 2.1.1. Patient Presentation

**Figure 1:**
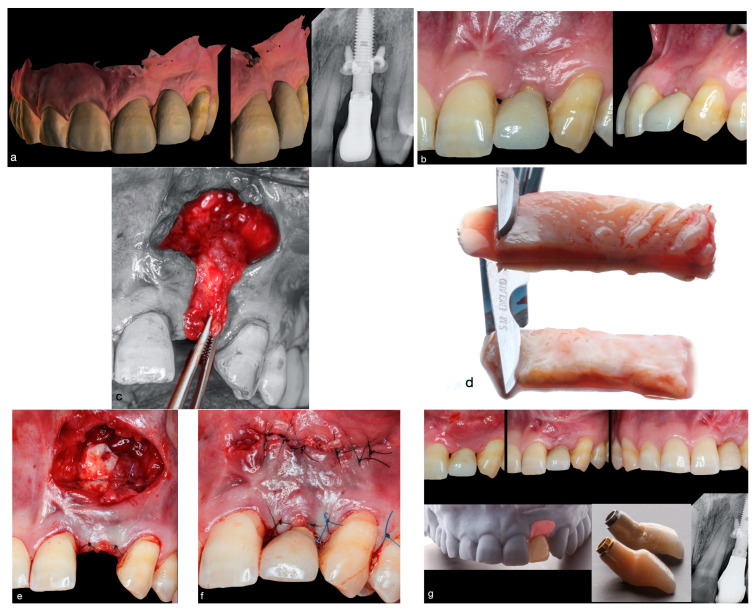
(**a**) Initial clinical situation. Periapical radiograph showing an excessively apical implant position. (**b**) Condition after modifying the cervical–buccal contour of the implant-supported crown. As well as the deficient MT, the partial loss of both papillae can be observed. (**c**) Dissection of the connective tissue pedicle graft. (**d**) Graft before de-epithelialization and graft after de-epithelialization with a 15C blade. (**e**) A CTG harvested from the palate was sutured over the connective tissue pedicle graft. (**f**) Flap closure. Suspension stitches were used in the marginal zone and simple stitches were placed without much tension to approximate the edges of the horizontal apical incision. (**g**) Healing 2 weeks (when stitches were removed), 1 month, and 4 months postsurgery (note the scar on the apical alveolar mucosa resulting from the surgical access incision). A cement- and screw-retained zirconia restoration with full reduction and ceramic stratification.

A 39-year-old man attended the Master’s in Regenerative Oral Surgery at the University of Salamanca, presenting with unfavorable esthetics concerning a restored implant in the left maxilla. The patient presented with no relevant medical history, and was a non-smoker. In the clinical examination of implant 22 (FDI numbering system), placed 3 years prior, extensive soft-tissue recession was observed due to a severe MT deficiency on the buccal of the implant, with partial loss of both peri-implant papillae (Figure 1a), whereas the KMW was satisfactory (≥2 mm KMW). Due to poor design of the cervical portion of the implant-supported restoration, the form of the peri-implant tissue had also been affected, with the peri-implant mucosal margin located apical to its ideal position. This resulted in patient dissatisfaction with the esthetics (Figure 1a). The patient presented a metal-ceramic screw-retained restoration on a unit transepithelial abutment with a Ti Golden^®^ surface.

The intraoral periapical radiograph (Figure 1a) showed the mesiodistal implant position to be correct, but the apico-coronal implant position was incorrect (overly deep implant placement, partially resolved with the transepithelial abutment), and the buccal–palatal plane was also incorrect (palatally positioned implant emergence). Two microscrews were also visible on the radiograph, suggesting that an earlier guided bone regeneration procedure had been performed on the buccal surface.

A prosthetic–surgical approach was therefore taken to treat the defect. The treatment objectives were to achieve the greatest possible KMW and thereby create a more favorable situation for peri-implant health, and to improve the esthetic appearance of the mucosal margin by replacing the implant crown.

The treatment plan was to modify the cervical contour of the existing implant-supported crown (Figure 1b) and, after 2 weeks, perform a bilaminar procedure using the modified apical access technique combined with a de-epithelialized connective tissue graft. After 3 months, a new implant-supported crown would be produced.

#### 2.1.2. Surgical Procedure

The surgical technique was performed following the description given by Quispe-López et al. [21] Prior to the surgical intervention, the crown supported by implant 22 was unscrewed, and the patient rinsed for 1 min with chlorhexidine mouthwash.

#### 2.1.3. Preparing the Recipient Site

**Figure 2:**
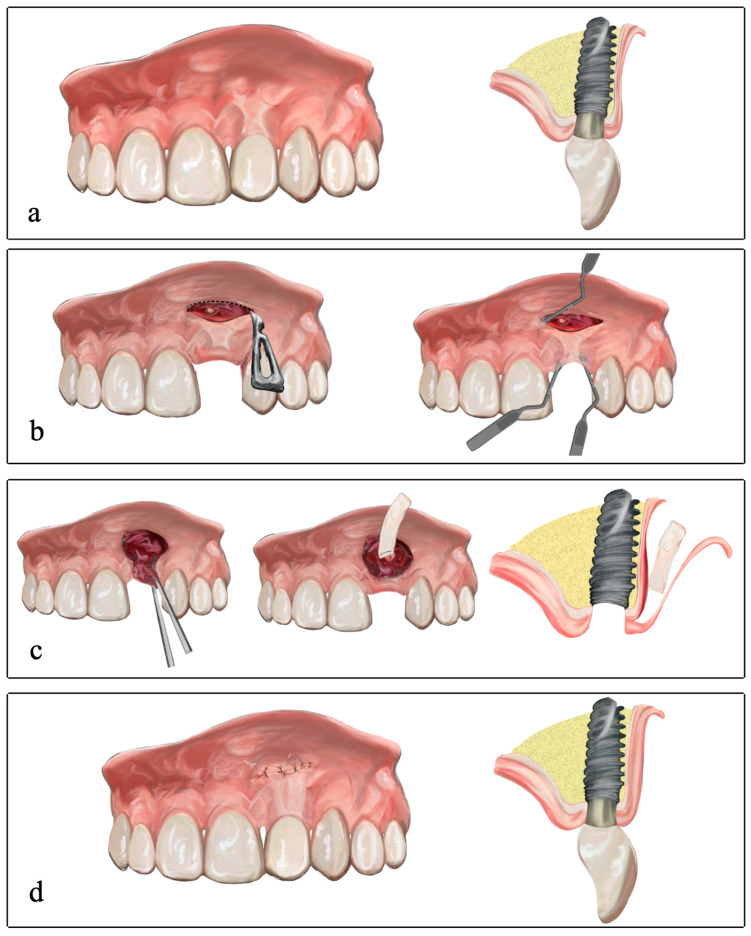
Schematic diagrams showing frontal and sagittal sections of the apical access technique with CTG. (**a**) Preoperative frontal and sagittal view of the mucosal defect. (**b**) Horizontal apical incision in the alveolar mucosa and tunneling of the recipient bed with instruments via the apical and marginal access. (**c**) Coronal advancement of the connective tissue pedicle graft and insertion of the de-epithelialized connective tissue graft through the horizontal apical incision. (**d**) Sagittal diagram in which the buccal MT gain is evident.

Following local anesthesia (using articaine 2% mg/mL with epinephrine 1:100,000), a single horizontal incision was made apical to the defect using a 15C blade (Swann-Morton, Owlerton Grn, Sheffield, UK). The incision was made partial-thickness on the buccal surface of the alveolar mucosa, and extended half a tooth mesiodistally on each side of the problematic site (middle of tooth 21 to the middle of tooth 23) (Figure 1c and Figure 2a–d). Using this incision, the flap was raised to partial thickness with a microscalpel (SB003 Spoon Blade, MJK instruments). Both papillae were included without cutting them, to leave a free and mobile flap (Figure 2b). In addition to the apical incision, an intrasulcular incision was made on the buccal aspect that extended from the papilla distal to 23 to the papilla mesial to 21, in order to connect and free the entire preparation on a single plane.

#### 2.1.4. Preparing the Connective Tissue Pedicle Graft

Apical to the first horizontal incision, at a distance (mm) equal to that between this incision and the mucosal margin of the implant crown, a horizontal incision was made in the periosteum and two slightly divergent vertical incisions were made in the coronal direction. A connective tissue pedicle graft was thereby created (composed primarily of periosteum and lamina propria) with its base apical to the first horizontal incision line. Once the connective tissue pedicle graft had been released, it was coronally advanced (Figure 1c and Figure 2c), adapted supraperiosteally, and sutured to the mesial and distal papillae using resorbable simple stitches.

#### 2.1.5. Harvesting the De-Epithelialized Free Gingival Graft

Next, a CTG was harvested from the palatal masticatory mucosa as a free gingival graft (FGG). A rectangular area was demarcated (two horizontal incisions and two vertical incisions) in the area of the palate between the distal aspect of the maxillary canine and the first molar. A partial-thickness graft was extracted containing epithelial tissue and connective tissue (FGG). After harvesting the FGG, it was extraorally de-epithelialized using a new 15C blade (Figure 1d).

#### 2.1.6. Graft Placement

The de-epithelialized free gingival graft (DFGG) was inserted through the buccal access at the back of the vestibule and adapted to the recipient site (Figure 2c). It is recommended to position the CTG over the pedicle flap made and sutured earlier (Figure 1e). The DFGG was fixed at the base of the papillae using two simple stitches (one simple stitch in the mesial papilla and the other in the distal papilla). To lightly compress the CTG, two simple stitches were then placed, using 5-0 resorbable sutures, anchored in the periosteum mesial and distal to the graft. The apical zone of the graft was fixed to the apical periosteum with another resorbable simple stitch to stretch the graft and prevent contraction. Six simple stitches were also used to close the buccal access incision (Figure 1f and Figure 2d). It is recommended that this incision be sutured so that it heals by primary intention, but excessive tension should not be generated, the aim being to approximate the wound edges without joining them closely together. Finally, two suspension stitches were used to coronally reposition the flap-CTG complex using 5-0 nonresorbable sutures (Figure 1f).

After the surgery, the patient received the anti-inflammatory medication dexketoprofen (Enantyum, Menarini; 25 mg tid for 5 days) and amoxicillin (Cinfa; 1 g bid for 7 days). The patient was advised not to brush the surgical area for 14 days after surgery and told to instead use a spray with 0.12% chlorhexidine + 0.05% cetylpyridinium chloride and a bioadhesive gel with 0.20% chlorhexidine and 0.20% hyaluronic acid (Perio-Aid, Dentaid) three times per day. At 4 weeks postsurgery, the patient started brushing again with a soft toothbrush using the roll technique. He was advised not to pull on his lip and to follow a bland, liquid diet for the first few days following surgery.

A check-up was made one week after the intervention, and sutures were removed after 14 days, when graft revascularization and epithelialization of the bloody area were observed. Evaluation and photographic follow-ups were performed after 1, 2 and 6 months of healing (Figure 1g).

#### 2.1.7. Digital Measurement of the Augmented Buccal Soft Tissue

The surgical site (implant 22) and its complete arch were scanned using an intraoral optical scanner (Trios, 3 Shape) at several points during the study: 4 weeks before surgery (T0), immediately before surgery (T1), and immediately (T2), 2 weeks (T3), and 6 months (T4) postsurgery. The digital models generated were exported and saved as STL (standard tesselation language) files, then imported into image analysis software (Geomagic Control X, 3D Systems, Rock, Hill, SC, USA). An examiner with extensive expertise in 3D volumetric assessment (TM) evaluated the changes in preoperative (T0 and T1) and postoperative (T2, T3 and T4) thickness. These changes in longitudinal thickness were examined on the mid-buccal aspect of the implant-supported crown using the 3D compare function which, after superimposing the models, enabled a color map to be created to quantitatively analyze the changes that had occurred in the areas of intervention. The color map ranges from +3 mm to −3 mm, with a tolerance of ±0.15 mm and is interpreted as follows: green areas show perfect alignment of the models; red, orange and yellow show an increase in volume; while dark and light blue represent a loss of volume. Next, a rectangular region of interest (ROI) was designed to study the linear changes in peri-implant mucosa in the area of intervention. Horizontally, the ROI included both papillae (mesial and distal), extending from the marginal contour of the implant crown to the marginal surface of the adjacent teeth. Positive values indicated that the peri-implant tissue was in a more buccal position (>thickness) while negative values showed that the peri-implant tissue was in a more palatal position (<thickness).

**Figure 3:**
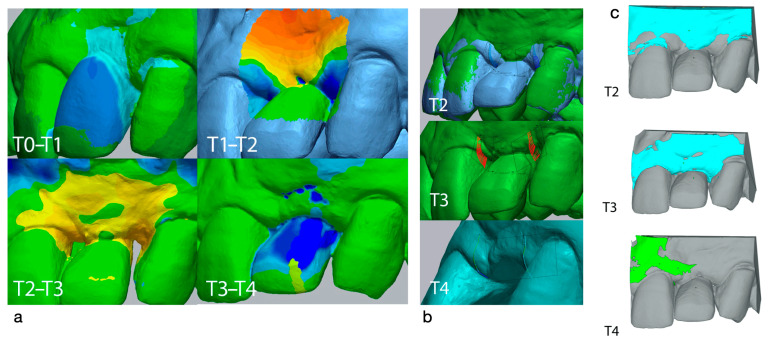
(**a**) Timeline of intraoral examinations divided into 4 main groups: T0–T1, T1–T2 (immediately before and immediately after the surgery), T2–T3 (immediately after the surgery and 2 weeks postsurgery) and T3–T4 (2 weeks and 6 months postsurgery). Each group describes a match between two neighboring STL datasets in the metrology software (Geomagic Control X). The tolerance threshold was set at ±0.1 mm. (**b**) Timeline of intraoral examinations divided into three main groups (T2, T3 and T4). The area examined (papillary complex) extended from the tip of the mesial and distal papillae to 3 mm apical. T3 shows the papilla augmentation in the T2–T3 period. (**c**) Region of interest with changes in volume measured in mm^3^. The T2 image shows the increase in volume (thickness) between T1 and T2. The T3 image shows the increase in volume (thickness) between T2 and T3, and the T4 image shows the minimal gain in volume from T3 to T4.

The soft tissue changes around the dental implant were demonstrated quantitatively and qualitatively (Figure 3a,b). The difference between T0 and T1 was not measured because there was a significant change in the crown contour, but a small reduction of thickness on the buccal contour was noted, with some migration of the tissue coronally (specifically, this migration was 1.62 mm between T0 and T1).

In the T1–T2 period, the average increase in MT was 0.78 mm, with a maximum increase of 2.68 mm. However, the papilla showed a slight decrease in height: 1.01 mm in the mesial papilla and 1.41 mm in the distal papilla. In the T2–T3 period, the average increase of thickness in the ROI was 0.84 mm, with a maximum increase of 1.47 mm. The mesial and distal papilla showed an increase in height of 1.62 mm and 1.655 mm, respectively (Figure 3a). In the T3–T4 period, the tissue was stable with a slight increase in thickness of 0.11 mm on average. For T4, the section measurement showed a slight average increase of 0.08 mm in the mesial papilla height, and a slight average decrease of 0.08 mm in the distal papilla height (Figure 3a). From T1 to T2, the volume of the ROI increased by 162.66 mm^3^. From T2 to T3 the volume increase was 73.99 mm^3^, while from T3 to T4 the volume increase was minimal, at only 3.63 mm^3^ (Figure 3b).

### 2.2. Case 2

#### 2.2.1. Patient Presentation

A 61-year-old man attended his annual check-up at the aforementioned clinic. The patient had no relevant systemic pathology and was a non-smoker. He presented with four implants in positions 24, 22, 11 and 14, placed five years earlier. He had been fitted with a Dolder bar retaining the four maxillary implants but, due to the problem with implant 22, multi-unit healing abutments had also been placed.

**Figure 4:**
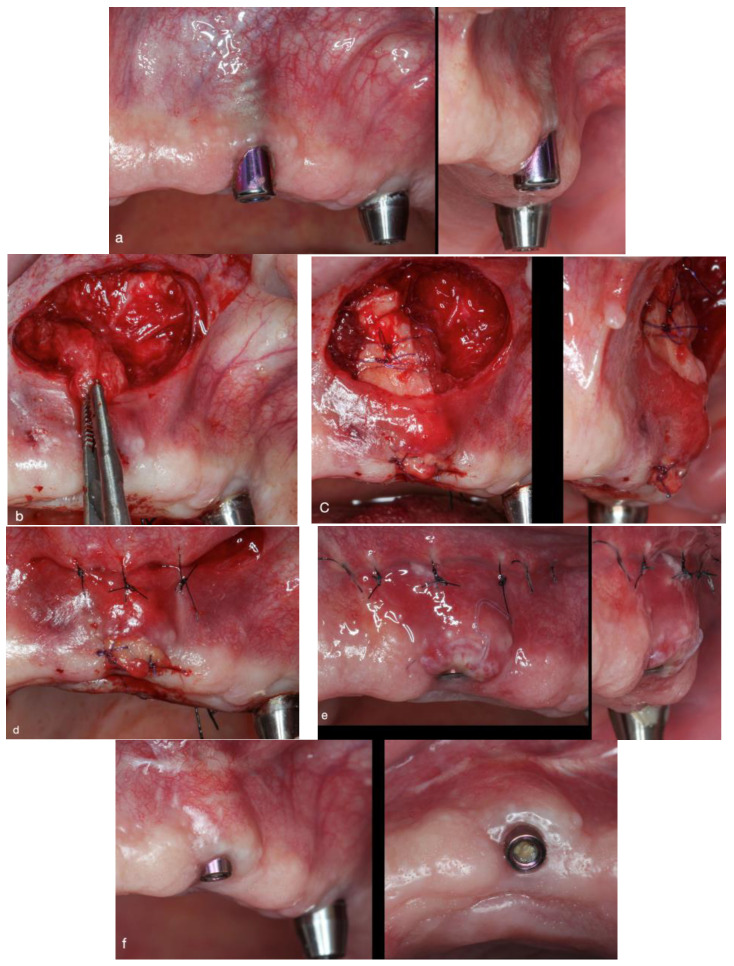
(**a**) Initial clinical situation, where the gray color of the implant 2.2 screw thread shows through the peri-implant mucosa. (**b**) Horizontal apical incision in the alveolar mucosa and dissection of the connective tissue pedicle graft. (**c**) Frontal and lateral view with the connective tissue pedicle graft + de-epithelialized connective tissue graft inserted into the recipient bed. (**d**) Flap closure with simple stitches using 6–0 monofilament. (**e**) Frontal and lateral view at 2 weeks postsurgery, when significant MT gain is evident. (**f**) Six months postsurgery; note the increase in MT and its stability after the 6-month follow-up.

In the clinical examination, inadequate MT and KMW (<2 mm) were observed buccal to implant 22, as was a complete lack of PBT, causing the implant screw thread to show through the buccal mucosa (Figure 4a). From a lateral viewpoint, the buccal implant position could be seen (implant malposition). The treatment plan was to take a purely surgical approach and, once the mucosal defect had been resolved, to reinsert the bar and overdentures. The treatment objective was to increase the KMW in order to improve peri-implant health and eliminate show-through of the implant screw thread.

#### 2.2.2. Surgical Procedure

To begin, the recipient bed was prepared (Video S1) by making a horizontal incision into the alveolar mucosa on the buccal aspect at the back of the vestibule using a 15C blade (Swann-Morton). The mesiodistal extension of the horizontal apical incision was as described in case 1 (it should be longer than the defect, extending approximately half a tooth beyond the defect mesially and distally).

From this horizontal incision, partial-thickness tunneling was performed. Full-thickness tunneling was only conducted in areas adjacent to the mucosal margin to minimize the risk of soft-tissue fenestration.

The connective tissue pedicle graft was then created, which involved rotating connective tissue and periosteum from the apical zone at the back of the vestibule toward the coronal zone (Figure 4b). To achieve this, 3 incisions were made (1 horizontal and 2 vertical) and the pedicled connective tissue was then coronally advanced and sutured at or past the mucosal margin using resorbable sutures.

After creating the recipient bed and suturing the connective tissue pedicle graft, a DFGG was harvested from the palatal masticatory mucosa. The DFGG was fixed onto the connective tissue pedicle graft with simple stitches using resorbable sutures (Figure 4c). Before closing the flap, plasma rich in growth factors (PRGF-Endoret System IV Biotechnology Institute) was applied to the recipient bed. The sutures were removed after 2 weeks, and the same postsurgical instructions were given as in case 1 (Figure 4d–f).

## 3. Discussion

In the current literature, a bilaminar approach (whether using a coronally advanced flap or tunneling flap) combined with a CTG is considered the most standardized and efficacious technique for treating peri-implant marginal mucosa defects (PMMDs) [22,23,24]. However, clinicians are increasingly choosing to make use of new approaches that forgo the classic access route. Research is therefore needed into techniques with remote access, such as the vestibular incision subperiosteal tunnel access technique (VISTA) [25] and apical approach surgical techniques [21]. The two clinical cases presented herein resulted in successful reconstruction of the peri-implant mucosa with a considerable increase in thickness, in clinical situations involving significant thinning of the buccal mucosa. This modification of the apical approach technique improves the vascularization of the recipient site due to the rotation of the CTG from the apical area, and secures a greater increase in MT through use of a DFGG from the palatal mucosa. In this context, creating a connective tissue pedicle graft from the zone apical to the defect is arguably less technically challenging, due to the location of the horizontal incision (at the back of the vestibule). However, for techniques requiring creation of a marginal access route, such as the coronally advanced flap, producing this inverted connective tissue pedicle graft would be more technically demanding, since it would necessitate making longer/deeper releasing incisions to properly access the apical zone at the back of the vestibule.

It should be noted that a purely surgical approach is not suitable for all clinical cases, as the prosthesis often needs to be replaced or modified (surgical-prosthetic approach) [26]. For example, in case 1, an extraoral modification of the facial contour of the existing prosthesis was performed 2 weeks before surgery, and the existing prosthesis was replaced by a new implant-supported crown two months postsurgery.

Harvesting CTGs from the palate is another clinical decision that is attracting increasing interest. The DFGG is now in much wider use than the subepithelial connective tissue graft (SCTG) [27,28]. In the cases described herein, the DFGG was chosen because it generally contains a larger amount of dense lamina propria and undergoes less contraction than the SCTG (greater adipose tissue content) [29,30].

In recent years, volumetric measurements based on conventional [19] or digital impressions [20,25] have made it possible to standardize MT measurement. In the present study on use of the modified apical access technique (connective tissue pedicle graft + DFGG) for peri-implant soft tissue augmentation, linear and volumetric measurements were assessed using an intraoral scanner (Trios, 3 Shape, A/S, Copenhagen, Denmark) and imaging software (Geomagic Control X, 3D Systems). In case 1, a total of five scans of the maxillary arch were performed at several points in the study (T0, T1, T2, T3 and T4), the STL files were then superimposed, and the changes analyzed using the imaging software. Our volumetric assessment showed an average MT gain of 0.84 mm in the T2 to T3 period, and practically no change between T3 and T4 (minimal MT gain: 0.11 mm on average). Our results support clinical studies showing greater increases in MT after use of a CTG. Nevertheless, it is important to remember that any type of graft (xenogeneic or autologous) results in a volumetric loss over a period of several months, this loss being greater in clinical situations where a CTG has not been performed [31].

To date, research on the apical approach technique for treating peri-implant soft tissue defects has been limited to case series [21]. The results obtained in these two clinical cases show a favorable outcome, with considerable MT gains that are important not only for treating unesthetic show-through of the implant screw thread, but also for improving esthetics and patient satisfaction in general. Further, the technique reduces invasiveness and minimizes tissue trauma, requiring only one horizontal incision in the apical area.

However, two limitations of the surgical technique should be noted. Firstly, the horizontal incision in the alveolar mucosa creates a small apical scar (this scar could be reduced through the use of materials such as plasma rich in growth factors or enamel matrix proteins). Secondly, in other clinical scenarios, when connective tissue was thin, it was not possible to prepare the connective tissue pedicle graft (Figure 1c and Figure 4b). In such situations, clinicians should therefore use the conventional technique [21].

More longitudinal clinical studies would be beneficial to analyze the efficacy of this modified technique for treating peri-implant soft tissue abnormalities and defects, in combination with digital volumetric analysis.

## 4. Conclusions

This article presents a modified approach to the apical access technique for treating significant deficiencies in MT. The modified technique obtained an increased blood supply in the recipient site (increased vascularization), larger gains in peri-implant mucosa and satisfactory esthetic results.

## Data Availability

The data presented in this study are available on request from the corresponding author.

## Supplementary Materials

The following supporting information can be downloaded at: https://www.mdpi.com/article/10.3390/dj12070194/s1, Video S1: clinical case 2: Modified apical access technique for the treatment of peri-implant soft tissue defects.

## Abbreviations

KMW	peri-implant keratinized mucosa width
MT	peri-implant mucosal thickness
STH	supracrestal tissue height
PBT	peri-implant bone thickness
CTG	connective tissue graft
DFGG	de-epithelialized free gingival graft
